# A New Tool Supporting the Selection of the Best Hematopoietic Stem Cell Donor by Modelling Local Own Real-World Data

**DOI:** 10.3390/jcm13226869

**Published:** 2024-11-15

**Authors:** Roberto Crocchiolo, Stefania Cacace, Giuseppe Milone, Barbara Sarina, Alessandra Cupri, Salvatore Leotta, Giulia Giuffrida, Andrea Spadaro, Jacopo Mariotti, Stefania Bramanti, Alice Fumagalli, Maria Pia Azzaro, Sebastiana Toscano, Quirico Semeraro

**Affiliations:** 1 Servizio di Immunoematologia e Medicina Trasfusionale, ASST Grande Ospedale Metropolitano Niguarda, 20162 Milano, Italy; 2 Dipartimento di Meccanica, Politecnico di Milano, 20156 Milano, Italy; stefania.cacace@polimi.it (S.C.); quirico.semeraro@polimi.it (Q.S.); 3 Ematologia, Azienda Ospedaliera Vittorio Emanuele, 95100 Catania, Italy; giuseppe.milone@gmail.com (G.M.); alessandracupri@hotmail.it (A.C.); leotta3@yahoo.it (S.L.); giulia.giuffrida@hotmail.it (G.G.); andrea.spadaro@hotmail.it (A.S.); mpazzaro@hotmail.com (M.P.A.); nellatosca@hotmail.it (S.T.); 4 Ematologia, Istituto Clinico Humanitas, 20089 Rozzano, Italy; barbara.sarina@humanitas.it (B.S.); jacopo.mariotti@humanitas.it (J.M.); stefania.bramanti@humanitas.it (S.B.); alice.fumagalli@st.hunimed.eu (A.F.)

**Keywords:** stem cell transplantation, donor, calculator, outcome

## Abstract

Background: The selection of the best donor for each specific patient is crucial for the success of allogeneic hematopoietic stem cell transplantation (HSCT). However, there is debate on the choice of the best donor when multiple suitable donors exist. Methods: By using own data from two transplant centers, we have developed a calculator able to provide the patients’ 2-year overall survival (OS) associated with each of the potential donor options during the selection process, in order to support the transplant physician during the choice. Data on 737 HSCTs with HLA-identical siblings, and unrelated or related haploidentical donors from January 2010 to July 2022 have been retrospectively obtained. Results: Patients’ age, disease, comorbidity index, and donor type were found to be significant variables able to predict the outcome with robustness (concordance index: 0.677). Estimates are provided within an example in the text showing outcomes with four donor options for a specific patient. Conclusions: We present the prototype of a tool supporting the selection of the best donor, guiding transplant physicians during the delicate process of donor selection before HSCT. This approach relies on real data from the centers, reflecting their local clinical experience. Improvements are underway with a larger, ongoing multicenter study.

## 1. Introduction

Allogeneic hematopoietic stem cell transplantation (HSCT) is a potential life-saving form of adoptive immunotherapy for both malignant and non-malignant disorders [1]. The hematopoietic stem cells are obtained from the bone marrow, peripheral blood, or umbilical cord blood of suitable donors, who are carefully selected by the transplant physicians according to patient, disease, and donor characteristics, according to clinical expertise and consensus guidelines. 

The selection of the most appropriate donor is critical for transplant outcome and the final choice relies on several factors, with the primary determinant in donor being human leukocyte antigen (HLA) patient–donor matching [2,3] and age thereafter [4,5,6]. HLA-identical sibling donors represent the first choice and further donor options are considered in patients who do not have such a related donor, as matched unrelated donors (MUDs), HLA-haploidentical related donors, and umbilical cord blood units represent the majority of donor sources altogether [1]. Besides HLA matching, additional factors, such as donors’ age, gender, ABO matching, and CMV serostatus, must be considered to ensure compatibility and minimize potential complications [3,7]. 

However, there is currently debate on the choice of the best stem cell donor when multiple suitable options exist. As an example, some reports suggest that a young unrelated donor might be preferred over an older HLA-identical sibling [8,9] or that a haploidentical related donor is comparable to matched, or even mismatched, unrelated one [10] in terms of the expected patient outcome after HSCT. Moreover, other donor-specific or patient–donor variables such as AB0-matching, gender parity for female donors, or cytomegalovirus serostatus are considered for the best donor selection, but a defined hierarchy is still lacking [4,11,12,13,14,15]. Nonetheless, these are all significant elements that contribute to patients’ outcome after HSCT. 

To address this issue, we have developed a calculator able to provide the patients’ 2-year overall survival (OS) associated with each of the potential donor options that physicians face during the selection process. All the available local own real data have been used to create predictive models that take into account the main patient, disease, transplant, and donor characteristics, focusing on the latter. By using real data from n = 737 HSCTs performed at two transplant centers, the tool allowed us to provide estimates associated with each potential donor type and thereby support the transplant physician during the delicate process of stem cell donor selection. 

## 2. Materials and Methods

### 2.1. Data Retrieval

We conducted a retrospective analysis on real-world data obtained from the clinical electronic charts of two transplant centers (Istituto Clinico Humanitas, Rozzano, Italy and Azienda Ospedaliera Vittorio Emanuele, Catania, Italy). The study was conducted on adult HSCTs performed between January 2010 and June 2022 by these two centers, who provided data and allowed for the creation of a unique database including patients’ variables, disease characteristics, transplant procedures, donor variables, and post-transplant outcomes. All these data are regularly provided by each transplant center active in Europe to the European Society for Blood and Marrow Transplantation (EBMT). All of the patients included signed a written informed-consent declaration and specific approval by the local Institutional Review Board was obtained. The data were divided into three time groups: at day 0 (patients’ variables, disease characteristics, transplant procedures, donor variables), day +100 (post-transplant outcomes), and annual follow-up (post-transplant outcomes). Only first HSCTs were considered, transplantations with multiple donors were excluded as well as those HSCT that were part of a planned multiple-graft protocol. The Day 0 requested variables were as follows: patient’s age and gender, date of initial diagnosis, primary disease at diagnosis, disease status at HSCT, comorbidity index, patient CMV status, HLA match, degree of mismatch, donor’s age and gender, donor CMV status, source of stem cells, graft manipulation, intensity of conditioning regimen, and graft-versus-host disease (GvHD) prophylaxis. The Day +100 requested variables were as follows: early graft loss, acute GvHD date of diagnosis and maximum extent, chronic GvHD date of diagnosis and maximum extent, first relapse or progression and date, survival status, and main cause of death. The requested variables for the annual follow-up were as follows: acute GvHD date of diagnosis and maximum extent, chronic GvHD date of diagnosis and maximum extent, late graft failure, first relapse or progression and date, survival status, and main cause of death. 

### 2.2. Statistical Analysis 

We developed a calculator, using real-world data from these two transplant centers, to estimate the 2-year OS for each potential donor during the selection process by investigating the significant factors influencing survival following HSCT. Parametric survival analysis was used to assess the relationship between the several covariates and the 2-year OS. Based on Anderson–Darling statistics, the Weibull distribution was selected as the most appropriate model for our data. This model enabled us to evaluate the impact of different variables on survival duration following HSCT. Both main effects and interactions were considered. The categorical variables assessed included diagnosis, HLA match, and the presence of comorbidities. The continuous variables assessed were the age of the patients and donors. Lastly, we evaluated interaction terms such as patients’ age with diagnosis, patients’ age with HLA match, and donors’ age with HLA match. Statistical significance was defined as *p*-value < 0.05, with 95% confidence interval. Minitab was used for statistical analyses (https://www.minitab.com/en-us/, accessed on 16 August 2024). After careful consideration, internal validation through the identification of a training and validation set was deemed unreliable due to relatively low numbers preventing us from creating a suitable model. As a consequence, a larger multicenter study was launched during the current analysis to improve the model and overcome this limitation by planning validation (see below). Finally, to assess the predictive quality of the model, a 5-fold cross-validation procedure was implemented. 

### 2.3. Calculator Output

The present calculator is intended to be a predictive tool to estimate the 2-year OS and the respective 95% confidence interval of each single patient according to a pre-selected stem cell donor, based on the actual current options during the selection process before HSCT. It utilizes a modelled database containing patient and donor information alongside post-transplant outcomes obtained from real-world, local clinical experience. By filtering the data from multiple donor options (i.e., an older HLA-identical sibling vs. a younger unrelated donor or an unrelated donor vs. a haploidentical) during the search, the tool provides the patient’s 2-year OS associated with each of these donors, thus supporting the selection of the best donor for that specific patient, according to the center’s experience. Importantly, a significant overlap of the outcome predictions between two or more donors may indicate that they are comparable in terms of the patient’s post-transplant survival and may suggest that those donors are equivalent; however, the calculator is intended to be a tool to support decision making but not be a decision-maker since the final choice will depend on multiple and somewhat complex and contingent factors. 

## 3. Results

### 3.1. Main Patient, Transplant and Donor Characteristics

The database originally contained n = 851 HSCTs reflecting the inclusion criteria. N = 114 were removed due to missing data, leading to n = 737 patients transplanted between July 2010 to January 2022 at the two above-cited transplant centers. N = 285 items were available for each patient. HSCTs from HLA-mismatched unrelated donors and cord blood units were excluded due to their limited numbers not allowing for a meaningful statistical analysis. The median patient and donor age at HSCT was 48 and 40, respectively. Male patients were n = 431 (58.5%) and female patients were n = 306 (41.5%). A total of n = 218 HSCTs were performed from HLA-identical siblings, n = 198 from matched unrelated donors (MUD), and n = 321 from haploidentical donors. Patients without comorbidities were n = 423 (57.4%), whereas n = 256 (34.7%) and n = 58 (7.8%) presented with 1 and ≥2 comorbidities, respectively. A total of n = 294 patients (39.9%) died following HSCT. Main transplant patient and donor characteristics are shown in Table 1. As one of the two centers is a well-recognized referral hospital for HSCT in lymphoma, diagnosis of Group 3 (see below) is slightly over-represented here compared to most of the series, where acute leukemias (Group 1) are usually predominant. 

GvHD prophylaxis was established according to the donor type and included high-dose post-transplant cyclophosphamide for recipients of haploidentical donors and anti-thymocyte globulin for recipients of matched unrelated ones, whereas no T-cell depletion was planned for transplants from HLA-identical siblings. 

### 3.2. Donor Type and Age 

**Figure 1:**
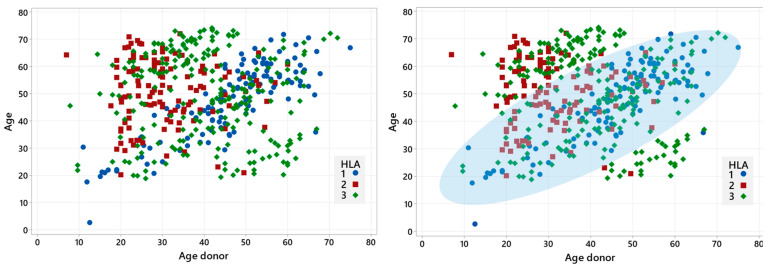
Scatterplots of patient and donors ages by HLA match types. The X axis represents the age of the donor, while the Y axis represents the age of the patient. Each point represents a donor–patient pair and is color-coded: HLA-identical sibling (blue), unrelated donor (red), haploidentical donor (green).

The type of stem cell donor and donor age are variables that are connected and not fully independent, due to three main reasons: (a) an HLA-identical sibling has usually approximately the same patient’s age; (b) a young MUD is mostly selected for donation; (c) a haploidentical donor is usually a sibling, a parent, or an offspring of the patient and therefore belongs to one of three age periods. The scatterplot in Figure 1 illustrates the correlation between the patients’ and donors’ age in the different HLA match categories, corresponding to HLA-identical siblings, MUD and haploidentical donors, respectively. The scatterplot on the right includes a density estimation providing a visual indication of where data points are concentrated. That is where the most common age combinations between patients and HLA-identical siblings, represented by blue dots, are found; as expected, the similar age distribution reflects the typical age gap between siblings. For MUD, depicted by red squares, the data are skewed towards younger ages, reflecting the broader age range found in unrelated donor registries. Haploidentical donors, represented by green diamonds, are broadly distributed but seem to be more concentrated in two points: there is a concentration of younger donors when the patients are older and, conversely, older donors when the patients are younger. This trend highlights the familial roles in donation as expected: older patients often have their children as donors, while younger patients frequently receive donations from their parents. 

### 3.3. Building the Model: Regression Analysis of Survival Predictors

The model fitted to the data is as follows [a]:

$$Y_{p}=\beta_{0}+\sum_{i=1}^{k}\beta_{i}x_{i}+\sum_{i<j}^{k}\beta_{ij}x_{i}x_{j}+\sigma \Phi^{-1}(p)$$

where *Y_p_* are the survival times (in days), *x_i_* (with *i* = 1, …, *k*) are the factors that are presumed to influence the survival times, and *β_i_* are the regression coefficients, *σ* is the shape parameter of the Weibull distribution, and $\Phi^{-1}(p)$ is the *p*th quantile of the standardized life distribution. 

Table 2 illustrates a regression analysis, based on the Weibull distribution, analyzing the impact of several variables, including the main factors (diagnosis, comorbidity, HLA match, patient’s age, and donor’s age) and interaction terms (Age patient × Diagnosis, HLA × Age patient, HLA × Age donor, Diagnosis × Age donor) on the 2-year OS following HSCT. Concerning the donor type (named here “HLA”), “HLA-1” is the reference category representing HLA-identical sibling, whereas “HLA-2” and “HLA-3” are MUD and haploidentical donors, respectively. The negative coefficients would suggest a lower risk for MUD and haploidentical donors compared to HLA-identical siblings. However, the *p*-values of 0.450 for MUD and 0.804 for haploidentical donors, indicate that these findings are not statistically significant. Only the patient age has a statistically significant *p*-value, indicating a slight increase in mortality risk as the age of the patient increases. 

Of note, the interaction term HLA × Age patient indicates how the effect of a patient’s age on HSCT outcomes varies according to the HLA match, that is the stem cell donor type. HLA-2 × Age patient (MUD) has a coefficient of −0.0269508 with a *p*-value of 0.003, which is statistically significant. The negative coefficient implies that as a patient’s age increases, the risk of negative outcomes is lower with an MUD compared to an HLA-identical sibling. HLA-3 × Age patient (haploidentical donors) has a coefficient of −0.01504909, but a *p*-value of 0.056, which is slightly above the threshold for statistical significance. This potentially suggests a decreased risk of negative outcomes with haploidentical donors compared to HLA-identical siblings as the patient’s age increases. Another important finding is the significant interaction term HLA × Age donor, indicating how the effect of a donor’s age on HSCT outcomes varies depending on the HLA match, that is the stem cell donor type. HLA-2 × Age donor has a coefficient of 0.0231561, while HLA-3 × Age donor has a coefficient of 0.0147905. The *p*-values are 0.020 and 0.046, indicating that both are statistically significant. The positive coefficients suggest that with increasing donor age, the survival following HSCT decreases, especially for HSCTs performed from MUD. 

**Figure 2:**
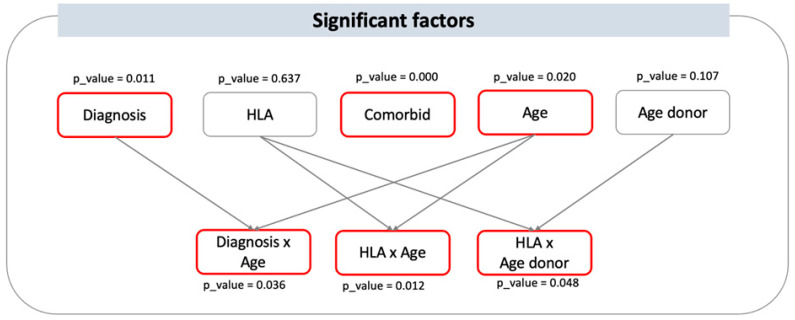
Significant factors influencing 2-year OS and their interactions. Visual representation of the statistically significant factors having an impact on 2-year OS following HSCT. Significant main variables are diagnosis, comorbidity, and age, with *p*-values of 0.011, 0.000, and 0.020, respectively. Donor type and donor age are not statistically significant as independent predictors of outcome; however, they are as interactions. Indeed, HLA × Age (i.e., patient age) and HLA × Age donor have *p*-values of 0.012 and 0.048, respectively, indicating that the effect of the patient and donor age depends on the HLA matching between patient and donor, that is here the donor type.

Overall, the significant factors having an impact on 2-year OS following HSCT are HLA × Age patient, diagnosis, HLA × Age donor, patient’s age, and Age patient × Diagnosis. The prediction of 2-year OS is deemed robust, with a concordance index of 0.677. The HLA match, that is the type of stem cell donor, demonstrates relevance to survival when analyzed in interaction with the patient’s age and the donor’s age, suggesting that the effect of age on outcome varies among the HLA match between patient and donor (Figure 2). Although disease stage at HSCT was included in the model, this variable failed to be statistically significant, likely due to the partial overlapping of “Diagnosis” and due to the relatively good outcomes in terms of 2-year OS for patients with lymphoproliferative and plasma cell disorders (here “Group 3”) who mostly underwent HSCT while not in remission, thus counterbalancing the favorable predictivity of complete remission status. Similarly, patient–donor sex mismatch has been analyzed but did not significantly correlate with 2-year OS in our series.

To assess the predictive quality of the model, a 5-fold cross-validation procedure was implemented [16]. The dataset was divided into five folds, aiming to achieve a balanced class distribution across each fold. In each iteration, the model was trained on four of the five folds and tested on the remaining fold, repeating this process five times to ensure robustness. The modified Brier Score for censored data [17,18] was used as the evaluation metric as it allows for an accurate assessment of predicted survival probabilities in the presence of censored cases. The Brier Score results for all validation runs are reported in Table 3. 

### 3.4. Example of Calculator

**Figure 3:**
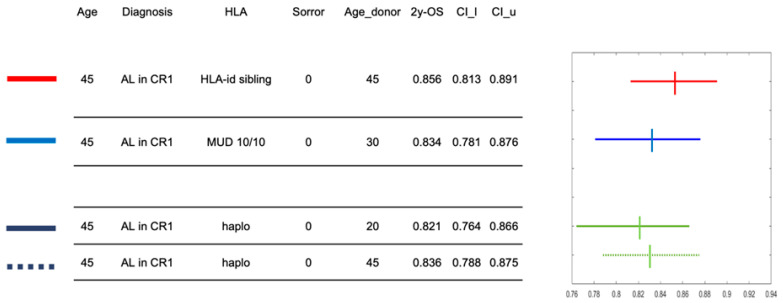
An example of calculator output for a defined patient and four stem cell donor options. The hypothetical patient is 45 years old and is affected by acute leukemia in first complete remission. There are four donor options during the search: a 45-year-old HLA-identical sibling, a 30-year-old unrelated donor, a 20-year-old haploidentical donor, a 45-year-old haploidentical donor. Hazard ratios of 2-year OS and 95% confidence intervals are shown on the right panel for each of the four donors.

Figure 3 reports an example of the calculator using a hypothetical, 45-year-old patient with a diagnosis of acute leukemia in first complete remission at HSCT and without any comorbidities (Sorror score of 0). Four possible donor options during the search occur: (a) a 45-year-old HLA-identical sibling; (b) a 30-year-old MUD; (c) a 20-year-old haploidentical donor; (d) a 45-year-old haploidentical donor. The calculator provides the 2-year OS of this patient associated with the four different donors and the graph on the right illustrates the confidence interval with the hazard ratio, providing guidance to the transplant physician in the decision-making process. As expected, the highest 2-year OS probability for this patient would be with a transplantation from an HLA-identical sibling, as it is 0.856. Estimates of 2-year OS following HSCT from either the MUD or the haploidentical donors are quite comparable, although with large 95% confidence intervals. These results inform the final choice of the best donor and are intended to integrate local selection algorithms and consensus guidelines. 

## 4. Discussion

Allogeneic hematopoietic stem cell transplantation is a complex therapeutic procedure, where patient-, disease-, transplant-, and stem cell donor-related variables determine the final outcome. As part of HSCT, the stem cell donor is usually selected by transplant physicians from multiple options that are carefully evaluated under local algorithms built on evidence from the literature and clinical experience.

We developed a calculator able to provide patient survival estimates associated with each one of the multiple donor options, in order to support the transplant physicians during the selection process. By modeling a dataset from n = 737 HSCTs from two centers, the proposed tool included the significant variables affecting post-transplant outcomes and relied on local own real-world data to provide 2-year OS estimates according to HLA-identical, unrelated, and haploidentical stem cell donor options. The tool calculates and prospectively suggests which would be the best donor based on the centers’ experience, since the results come from a model based on real data of the same centers, representing a novel and original element of our proposed tool. To our knowledge, at least two calculators have already been developed; however, they use aggregated data from the registry and were not tailored to donor selection among multiple donor types [19,20]. Moreover, there is currently no specific study focusing on donor selection and donor characteristics across all transplant types, which is the focus of our study, taking into account HLA-identical siblings, unrelated donors, and haploidentical donors at the same time. Although the choice of the stem cell donor is mainly based on HLA matching and donor age, our model demonstrates that these two variables interact and that the impact of HLA matching varies according to both patient and donor age. This is in line with the evidence that young unrelated donors are preferred over older ones and might be a better option when compared with older HLA-identical siblings [9]. Moreover, a timely haploidentical donor may be a suitable option if an unrelated donor requires a long delay, negatively affecting patient prognosis, or whether an HLA-mismatched unrelated donor is solely available [21]. For these reasons, such a calculator may be useful to support decision making among multiple donor options and it is worth noting that the modeling comes from local own real data, reflecting the clinical experience of the center.

We show here the feasibility of developing such a tool that may help the transplant physicians in the decision-making process of selecting the most suitable stem cell donor for transplantation, even when the results between potential donors are statistically similar. In this latter case, for example, the final donor choice may rely on other elements, such as any urgent timing needed to proceed to HSCT, better donor availability, or the expected compliance to stem cell mobilization and collection. These points are equally critical for the success of HSCT and are regularly considered during selection in real-life settings; therefore, similar expected 2-year survival outcomes associated with two or three donor options might guide the transplant physician to mainly address these other elements in the final donor selection. Our main purpose was to effectively use real data information by comparing the survival outcomes associated with different donors, to inform for a hopefully optimal selection.

We acknowledge some limitations, such as missing data and the final number of HSCTs, preventing more definite estimates and narrower confidence intervals; similarly, internal validation was initially planned but was showed to be unfeasible because it would have drastically reduced the training set size, compromising the model quality due to the limited data available for estimating parameters, especially given the high number of factors under consideration. In addition, we cannot exclude that the partial knowledge of the complex inter-relation between variables, the existence of unknown variables, or of variables not captured by current models, may have affected the performance of our model. However, since the main aim of the present study was to build a prototype calculator, we focused on a few transplant centers (namely two) and believe that our scope has been fulfilled, showing the feasibility of this approach and the novelty of including different donor types (HLA-identical siblings, MUD, haploidentical) in the same model. Moreover, we explored multiple and significant interactions between variables, and this probably deserves further investigation since most regression models do not frequently include interaction terms, although they may be clinically meaningful. Nonetheless, a prediction of 2-year OS is deemed robust here, with a concordance index of 0.677, thus deserving further development with larger datasets. Moreover, internal cross-validation showed satisfactory predictive performance as indicated by the average Brier Score (see Table 3), especially considering the relatively limited sample size. Of course, caution is needed when extrapolating our results due to the lack of internal or external validation, as previously stated; nonetheless, our calculator represents a first attempt to provide prospective suggestion on the best stem cell donor during the delicate process of selection by providing the patient’s survival estimates associated with each of the donor options, with the aim of supporting the final donor selection, that represents a critical step operated by the transplant physicians according to their own experience and to consensus guidelines. Some unusual findings from our prior example (i.e., lower survival hazard ratio with younger haploidentical donors) might be due to the number of HSCTs here preventing narrower confidence intervals. For these reasons, we are moving forward with an ongoing multicenter study with over 10,000 HSCTs, having the aim of fine-tuning the predictive model by increasing the total numbers of HSCTs and the variables/interactions, performing validation and including machine-learning approaches. This next step will hopefully provide a ready-to-use calculator with wider applicability, also taking into account any differences between centers due to demographics, protocols, and clinical expertise.

In conclusion, the present study showed the feasibility of using transplant centers’ retrospective data to prospectively suggest the best stem cell donor during the delicate process of selection, according to the center experience. We believe that this approach improves performance and applicability over external models since our results are derived from local own real-world data information and take into account multiple distinct donor types (HLA-identical sibling, unrelated, and haploidentical donors) at the same time.

A collaborative multicenter study is underway with the aim of increasing the number of transplants, refining the impact of variables and their interactions, and performing internal validation, to finally improve the model’s performance.

## Figures and Tables

**Table 1:** Main patients and donor characteristics.

Patient Characteristics	Number of Patients	Identical Sibling	Matched Unrelated	Haploidentical
Number of patients	737	218	198	321
Gender				
Male	431	130	112	188
Female	306	88	86	133
Median age at transplantation (range)	48 (21–71)	48 (21–71)	50 (22–71)	49 (21–68)
HLA match				
Identical sibling	218	218	-	-
Matched unrelated	198	-	198	-
Haploidentical	321	-	-	321
Diagnosis *				
Group 1	306	107	96	102
Group 2	114	26	44	44
Group 3	317	85	58	175
Median Karnofsky score	87.7	85.4	88.4	89.7
Karnofsky score >90% at HSCT	446	105	129	212
Positive CMV serostatus	684	228	171	285
Negative CMV serostatus	53	15	18	20
Total comorbidities				
No comorbidity	423	152	110	161
1 comorbidity	256	51	71	134
>2 comorbidities	58	15	17	26
**Donor Characteristics**	**Number of Donors**	**Identical Sibling**	**Matched Unrelated**	**Haploidentical**
Median age at donation (range)	40 (19–58)	46 (30–57)	32 (19–48)	41 (19–58)
Gender				
Male	500	145	151	204
Female	231	72	47	112
missing	6	1	0	5
Positive CMV serostatus	556	183	128	245
Negative CMV serostatus	181	35	70	76

* Patients’ diagnoses were categorized based on similar biologic characteristics of the disease, given the limited number of patients with individual diagnoses: group 1 = acute undifferentiated leukemia, acute myeloid leukemia and related precursor neoplasms, mixed phenotype acute leukemia, mixed phenotype B/myeloid, secondary acute leukemia, precursor lymphoid neoplasms; group 2 = chronic leukemia, chronic myeloid leukemia, myeloproliferative neoplasia, myelodysplastic syndrome or myeloproliferative syndrome, myelodysplastic syndrome, myelodysplastic and myeloproliferative syndromes; group 3 = Hodgkin’s lymphoma, chronic lymphocytic leukemia, Non-Hodgkin’s lymphoma, lymphoma (not otherwise specified), prolymphocytic leukemia, multiple myeloma, plasma cell leukemia. Numbers of individual diagnoses are as follows: acute undifferentiated leukemia n = 2, acute myeloid leukemia and related precursor neoplasms n = 214, mixed phenotype acute leukemia n = 1, mixed phenotype B/myeloid n = 2, secondary acute leukemia n = 16, precursor lymphoid neoplasms n = 71 (total Group 1 n = 306); chronic leukemia n = 1, chronic myeloid leukemia n = 5, myeloproliferative neoplasia n = 26, myelodysplastic syndrome or myeloproliferative syndrome n = 1, myelodysplastic syndrome n = 67, myelodysplastic and myeloproliferative syndromes n = 14 (total Group 2 n = 114); Hodgkin’s lymphoma n = 136, chronic lymphocytic leukemia n = 10, Non-Hodgkin’s lymphoma n = 138, lymphoma (not otherwise specified) n = 1, prolymphocytic leukemia n = 1, multiple myeloma n = 29, plasma cell leukemia n = 2 (total Group 3 n = 317).

**Table 2:** Variables affecting 2-year OS after HSCT and their interactions.

Predictor	Coefficient	Standard Error	Z Value	*p* Value	Lower CI	Upper CI
Intercept	7.35820	0.239108	30.77	0.000	6.88956	7.82684
Diagnosis_1 *						
2	1.23406	0.516391	2.39	0.017	0.221949	2.24616
3	0.603906	0.271176	2.23	0.026	0.0724101	1.13540
HLA_1 **						
2	0.267212	0.354110	0.75	0.450	−0.426830	0.961255
3	−0.0726015	0.292851	−0.25	0.804	−0.646578	0.501375
Age patient	0.0164252	0.0076519	2.15	0.032	0.0014279	0.0314226
Age donor	−0.0090390	0.0077637	−1.16	0.244	−0.0242556	0.0061776
Age_patient × Diagnosis_1						
2	−0.0189244	0.0079260	−2.39	0.017	−0.0344590	−0.0033898
3	−0.0055234	0.0050316	−1.10	0.272	−0.0153852	0.0043383
HLA_1 × Age patient						
2	−0.0269508	0.0092053	−2.93	0.003	−0.0449929	−0.0089097
3	−0.0150409	0.0078692	−1.91	0.056	−0.0304643	0.0043383
HLA_1 × Age donor						
2	0.0231561	0.0099341	2.33	0.020	0.0036856	0.0426266
3	0.0147905	0.0074005	2.00	0.046	0.0002859	0.0292951
Diagnosis_1 × Age donor						
2	−0.0045711	0.0072265	−0.63	0.527	−0.0187347	0.0095926
3	−0.0023857	0.0050394	−0.47	0.636	−0.0122628	0.0074914
Shape	1.69843	0.0654468			1.57488	1.83167

* Diagnoses were grouped according to what already described in Table 1. ** HLA_1 = HLA-identical sibling; HLA_2 = unrelated donor; HLA_3 = haploidentical donor.

**Table 3:** Results of 5-fold Cross validation in terms of modified Brier Score for censored data.

Validation Run	BR Score
1	0.313
2	0.303
3	0.244
4	0.334
5	0.290
Mean ± 1 st.dev	0.297 ± 0.03

[16,17,18].

## Data Availability

Anonymous and aggregated data could be made available upon reasonable request and after authors’ approval. Personal data protection: https://www.garanteprivacy.it/home/docweb/-/docweb-display/docweb/9124510 (accessed on 29 October 2024).
